# Development and Validation of an Immune-Related Prognostic Signature for Laryngeal Squamous Cell Carcinoma

**DOI:** 10.3390/jcm15145382

**Published:** 2026-07-09

**Authors:** Changding He, Wanqiu Peng, Yi Shi, Huaidong Du

**Affiliations:** 1 Department of Otorhinolaryngology, Eye & ENT Hospital, Fudan University, Shanghai 200031, China; hechangding@163.com; 2 Zhongshan Hospital, Fudan University, Shanghai 200032, China; peng.wanqiu@zs-hospital.sh.cn; 3 Department of Clinical Laboratory, Punan Branch of Renji Hospital, Shanghai Jiao Tong University, Shanghai 201312, China

**Keywords:** laryngeal squamous cell carcinoma, immune-related genes, risk stratification, precision oncology

## Abstract

**Background**: Laryngeal squamous cell carcinoma (LSCC) is a highly aggressive malignancy with poor prognosis, particularly in advanced stages. While traditional treatments have improved survival rates, reliable biomarkers for prognosis remain limited. **Methods**: We analyzed RNA-seq data of LSCC patients from the Cancer Genome Atlas (TCGA) and validated the results using the Gene Expression Omnibus (GEO) dataset (GSE27020), clinical samples, and LSCC cell lines. Differentially expressed immune-related genes (DEIRGs) were identified using the “limma” R package. A prognostic signature was developed by integrating univariate Cox analysis, least absolute shrinkage and selection operator (LASSO) regression, and multivariate Cox analysis. The signature’s predictive performance was validated using Kaplan–Meier survival analysis and receiver operating characteristic (ROC) curves. **Results**: A three-gene immune-related prognostic signature comprising TNFRSF4, PPARG, and PDGFA was established. In the training cohort, the model stratified patients into high- and low-risk groups with significantly different overall survival (HR = 5.81, 95% CI: 2.56–13.22, *p* < 0.001), with apparent 1-, 2-, and 3-year AUC values of 0.838, 0.895, and 0.947, respectively. Predictive performance was further evaluated in the TCGA testing cohort, the full TCGA cohort, and the GSE27020 cohort. Functional enrichment analysis revealed that the signature genes are involved in immune regulation and tumor progression. **Conclusions**: This study identified and validated a novel three-gene immune-related prognostic signature for LSCC, offering a practical tool for individualized prognosis and personalized treatment strategies. The signature provides insights into immune-related mechanisms in LSCC, presenting potential targets for therapeutic intervention.

## 1. Introduction

Laryngeal squamous cell carcinoma (LSCC) accounts for a major fraction of head and neck malignancies and is frequently associated with substantial morbidity and mortality. Most tumors arise in the glottic region, with the remainder originating from supraglottic or subglottic subsites [1]. Established risk factors include tobacco exposure, alcohol consumption, air pollution, human papillomavirus infection, and chronic inflammation [2]. Despite advances in surgery, radiotherapy, chemotherapy, and organ-preserving strategies, outcomes for patients with advanced disease remain unsatisfactory [3]. More precise biomarkers are therefore needed to complement anatomical staging and improve prognostic assessment in LSCC.

Tumor immunity is a central determinant of cancer progression, immune escape, and therapeutic response. Immune checkpoint blockade has reshaped the treatment landscape for several malignancies, including recurrent or metastatic head and neck squamous cell carcinoma (HNSCC) [4,5,6]. In HNSCC, the tumor immune microenvironment reflects reciprocal interactions among malignant epithelial cells, infiltrating immune cells, fibroblasts, endothelial cells, and extracellular matrix components. These interactions can either sustain antitumor surveillance or create an immunosuppressive niche that supports tumor growth and treatment resistance [7].

Several immune-related expression models have been proposed for HNSCC. For example, immune-related gene-pair and innate-immunity gene-pair signatures have shown prognostic value in TCGA and GEO datasets and have been associated with survival outcomes and immune status [8,9]. However, HNSCC is biologically heterogeneous, and models trained across mixed anatomical subsites may not fully capture the immune biology of LSCC. Prognostic tools developed specifically for LSCC remain limited.

A more focused characterization of LSCC-associated immune genes could therefore refine survival stratification and generate biologically grounded hypotheses about the tumor microenvironment. Such work is particularly relevant as immunotherapy moves from recurrent or metastatic disease toward earlier clinical settings, where patient selection and risk assessment remain unresolved.

Here, we integrated LSCC transcriptomic profiles with curated immune-related genes to develop a three-gene prognostic signature. We assessed the model in internal and external cohorts, examined its independence from available clinicopathological variables, and evaluated expression of the signature genes in paired clinical samples and cell lines.

## 2. Materials and Methods

### 2.1. Public Datasets

RNA-sequencing data from 111 LSCC tissues and 12 paired adjacent tissues were obtained from TCGA (https://portal.gdc.cancer.gov; accessed on 15 March 2023) [10]. Available clinicopathological information, including age, gender, tumor grade and stage, was also retrieved (Supplementary Table S1). Treatment-related variables were reviewed where available, but detailed treatment modality, surgical margin status, extranodal extension, tumor subsite and HPV/p16 status were incomplete and were not included in the primary multivariable model. The GSE27020 cohort (*n* = 109), generated on the GPL96 platform (Affymetrix Human Genome U133A Array), was downloaded from GEO and used for external validation (Supplementary Table S4) [11,12].

### 2.2. Identification of Immune-Related Genes

A curated list of 2498 immune-related genes was obtained from ImmPort (https://www.immport.org; accessed on 15 March 2023). TCGA RNA-sequencing data were analyzed in R (version x64 3.6.1). Differentially expressed immune-related genes (DEIRGs) were identified using the limma R package (version 3.42.2), with |log2 fold change| ≥ 0.5 and adjusted *p* < 0.05 as thresholds.

### 2.3. GO and KEGG Enrichment Analysis

To examine the biological context of the DEIRGs, we performed Gene Ontology (GO) and Kyoto Encyclopedia of Genes and Genomes (KEGG) enrichment analyses across biological process, molecular function and cellular component categories [13,14,15]. Subnetwork enrichment results are provided in Supplementary Table S2.

### 2.4. Protein–Protein Interaction (PPI) Network

DEIRGs with a STRING interaction score greater than 0.4 were included in the protein–protein interaction (PPI) analysis [16]. Networks and subnetworks were visualized and annotated using Cytoscape (version 3.7.1; Cytoscape Consortium, San Diego, CA, North America, USA) [17].

### 2.5. Establishment of the IRG-Associated Prognostic Signature

TCGA samples were randomly divided into a training cohort (*n* = 56) and a testing cohort (*n* = 55). In the training cohort, univariate Cox proportional hazards regression was used to screen immune-related genes associated with overall survival. LASSO Cox regression was then applied for dimensionality reduction, with the penalty parameter selected by 10-fold cross-validation to limit model complexity. Genes with non-zero coefficients were entered into multivariable Cox regression to derive the final risk-score model.

The risk score was calculated as follows: (−1.175739543 × TNFRSF4 expression) + (1.214802258 × PPARG expression) + (1.011881540 × PDGFA expression). Patients were assigned to high- or low-risk groups using the median risk score in the training cohort. The same formula and cut-off strategy were applied to the testing cohort, the full TCGA cohort and GSE27020. Survival differences were tested using Kaplan–Meier curves and log-rank tests, and discrimination was quantified using time-dependent ROC analysis.

### 2.6. Cox Regression Analysis

Univariate and multivariable Cox regression analyses were performed in the full TCGA cohort to determine whether the signature was independently associated with overall survival. Available covariates were age, gender, tumor grade and stage.

### 2.7. Construction of a Nomogram Based on the IRG-Based Signature

An exploratory nomogram was built from the risk score and available clinical variables (age, gender, grade and stage). Its performance was evaluated using calibration plots and time-dependent ROC curves.

### 2.8. Ethics Statement and Tissue Specimens

Thirty paired LSCC and adjacent non-tumor tissue samples were collected between September 2020 and July 2021 at the Department of Otorhinolaryngology Head and Neck Surgery, Eye and ENT Hospital, Fudan University, to validate the differential expression of signature genes. All participants provided written informed consent. The study was approved by the institutional ethics committee (approval code: 2021039) and conducted in accordance with the Declaration of Helsinki. Clinicopathological characteristics of the qRT-PCR cohort, including age, gender, smoking status, alcohol consumption, HPV status, primary site, TNM stage and treatment regimen, are summarized in Supplementary Table S5.

### 2.9. Cell Culture

The LSCC cell lines AMC-HN8 and Tu686 were used for in vitro expression analysis. Tu686 cells were cultured in DMEM (Gibco), and AMC-HN8 cells were cultured in RPMI-1640 (HyClone). Both media were supplemented with 10% fetal bovine serum (Gibco), and cells were maintained at 37 °C in 5% CO_2_. HuLa-PC, a laryngeal epithelial cell line derived from the posterior commissure, was obtained from ATCC (Gaithersburg, MD, USA) and cultured in Dermal Cell Basal Medium (ATCC PCS-200-030).

### 2.10. qRT-PCR

Total RNA was extracted from tissues and cell lines using TRIzol reagent (15596018, Invitrogen, Thermo Fisher Scientific, Waltham, MA, USA). RNA was reverse-transcribed with the Evo M-MLV RT Kit with gDNA Clean for qPCR (AG11711; Accurate Biology, Changsha, China). qRT-PCR was performed using the SYBR Green Premix Pro Taq HS qPCR Kit (AG11701; Accurate Biology, Changsha, China) on an ABI 7500 Real-Time PCR System (Applied Biosystems, Foster City, CA, USA). GAPDH was used as the endogenous control. Primers were synthesized by Sangon Biotech (Shanghai, China), and their sequences are listed in Supplementary Table S3.

### 2.11. Statistical Analyses

Statistical analyses were performed using R (version 3.6.1; R Foundation for Statistical Computing, Vienna, Europe, Austria) and GraphPad Prism (version 7.0; GraphPad Software Inc., San Diego, CA, USA). p values for differential expression were adjusted using the Benjamini–Hochberg false discovery rate. Cox models are reported as hazard ratios (HRs) with 95% confidence intervals (CIs). Kaplan–Meier curves and log-rank tests were used for survival comparisons, and time-dependent ROC analysis evaluated discrimination at 1, 2 and 3 years. For external validation in GSE27020, microarray probes were mapped to gene symbols. Student’s *t*-test was used to compare two groups when the data followed a normal distribution; otherwise, the nonparametric Mann–Whitney U test was used. A *p* value < 0.05 was regarded as statistically significant.

## 3. Results

### 3.1. Identification of Immune-Related Genes

**Figure 1:**
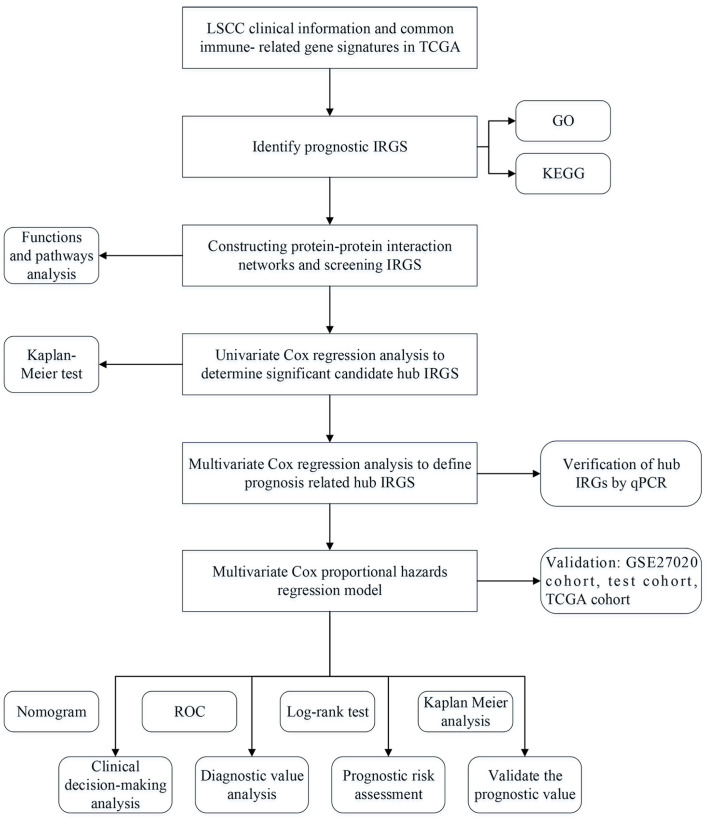
Study workflow. TCGA samples were divided into training (*n* = 56) and testing (*n* = 55) cohorts; the full TCGA cohort (*n* = 111) and GSE27020 (*n* = 109) were used for model evaluation.

**Figure 2:**
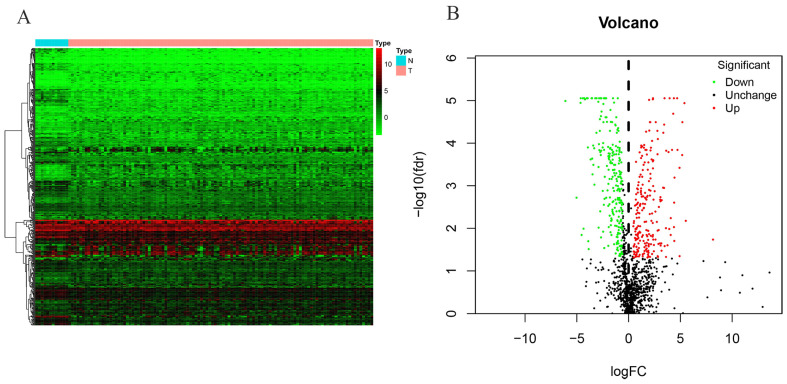
Differential expression of immune-related genes. (**A**) Heatmap of the 50 most significant DEIRGs. (**B**) Volcano plot showing upregulated and downregulated IRGs.

The study workflow is shown in Figure 1. A total of 481 differentially expressed immune-related genes (DEIRGs) were identified based on the screening criteria (|log2(FC)| ≥ 0.5 and adjusted *p*-value < 0.05). The top 50 DEIRGs with the most significant adjusted *p*-values are presented in a heatmap in Figure 2A, while the distribution of these DEIRGs is visualized in a volcano plot in Figure 2B. These visualizations collectively highlight the significant differences in gene expression profiles between normal and tumor tissues, providing a set of potential candidate genes for further investigation.

### 3.2. Functional Enrichment Analysis

**Figure 3:**
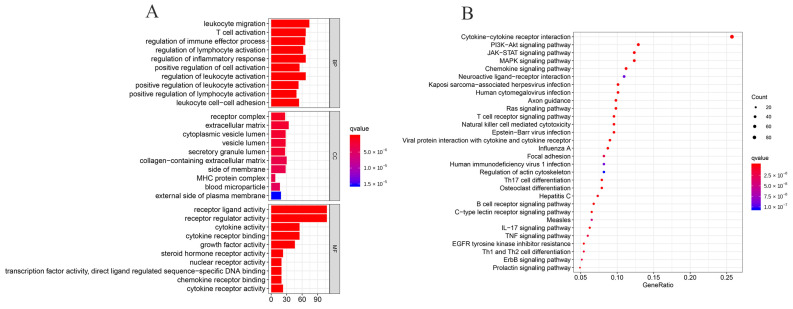
Functional enrichment of DEIRGs. (**A**) Significantly enriched GO terms. (**B**) Top 30 significantly enriched KEGG pathways.

Functional enrichment analysis of DEIRGs revealed distinct immunological signatures (Figure 3). GO analysis showed that these genes are mainly involved in immune-related biological processes such as leukocyte migration, T cell activation, regulation of immune effector processes, and inflammatory response. In terms of molecular function, receptor ligand and regulator activities were significantly enriched. KEGG pathway analysis further identified enrichment in cytokine-cytokine receptor interaction, PI3K-Akt, JAK-STAT, MAPK, and chemokine signaling pathways, along with others related to T cell and natural killer cell activity. Together, these results indicate that DEIRGs in LSCC are primarily associated with immune regulation and signal transduction, highlighting key mechanisms of disease pathophysiology and suggesting potential therapeutic targets.

### 3.3. PPI Network Construction

**Figure 4:**
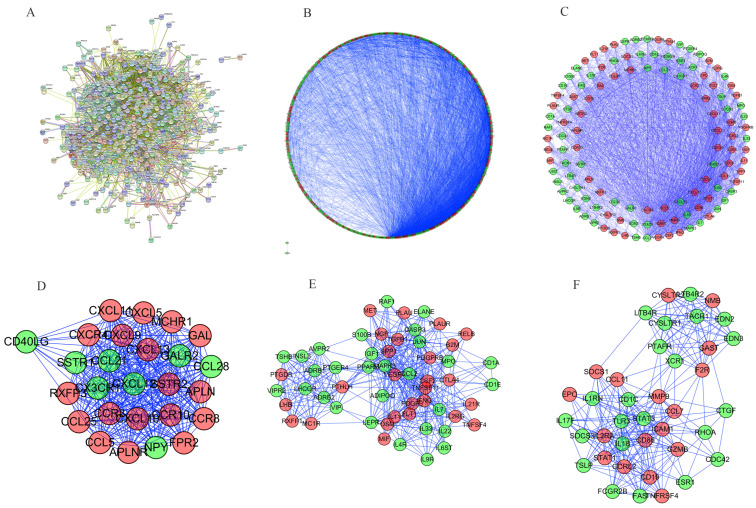
PPI analysis of DEIRGs. (**A**,**B**) Network visualizations. (**C**–**F**) Leading subnetworks identified from the PPI network. In B–F, red and green nodes indicate up- and downregulated DEIRGs, respectively.

To elucidate the functional relationships among DEIRGs, we constructed a comprehensive protein–protein interaction (PPI) network (Figure 4A), which revealed extensive interconnectivity among immune mediators. Visualization using circular and bipartite layouts (Figure 4B,C) highlighted the distribution of upregulated and downregulated genes within the network. Modular analysis identified three key subnetworks: a chemokine/cytokine module centered on CXCL family members (Figure 4D), a growth factor and receptor tyrosine kinase module with multiple interleukins and growth factors (Figure 4E), and a cell adhesion/migration module involving integrin-associated proteins (Figure 4F). Notably, hub genes such as CXCL8, IL1B, and STAT1 showed high connectivity, indicating their central roles in immune regulation. These findings provide mechanistic insight into the molecular architecture and immune microenvironment dysregulation in LSCC.

### 3.4. Identification and Verification of the IRG-Associated Prognostic Signature

**Figure 5:**
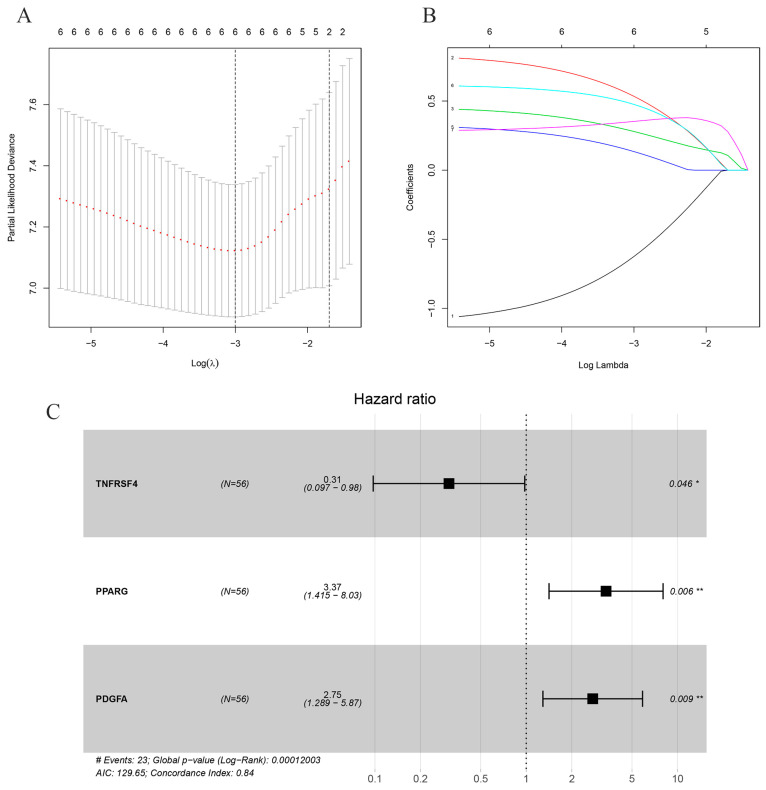
Construction of the prognostic model. (**A**) Cross-validation for λ selection; red dots and gray error bars indicate the mean cross-validated partial likelihood deviance and ±1 standard error, respectively, and vertical dotted lines mark λ_min and λ_1SE. (**B**) LASSO Cox coefficient profiles; colored curves denote gene-specific coefficient trajectories as log(λ) changes. (**C**) Multivariable Cox regression forest plot. * *p* < 0.05; ** *p* < 0.01.

To identify prognostic immune-related genes, we applied univariate Cox analysis followed by LASSO Cox regression on the training cohort (Table 1). Using 10-fold cross-validation, the penalty parameter corresponding to the minimum cross-validated partial likelihood deviance (λ = 0.001) was selected. Given the limited sample size, this value provided only minimal coefficient shrinkage and should be primarily interpreted as a feature-selection step rather than strong regularization (Figure 5A,B). This procedure identified three genes with non-zero coefficients: TNFRSF4, PPARG, and PDGFA. Multivariate Cox regression showed that TNFRSF4 was associated with favorable prognosis (HR = 0.31, 95% CI: 0.10–0.98, *p* = 0.046), whereas PPARG (HR = 3.37, 95% CI: 1.42–8.03, *p* = 0.006) and PDGFA (HR = 2.75, 95% CI: 1.29–5.87, *p* = 0.009) were associated with poorer prognosis (Figure 5C, Table 2).

### 3.5. Validation and Performance Assessment of the Three-Gene Prognostic Model

**Figure 6:**
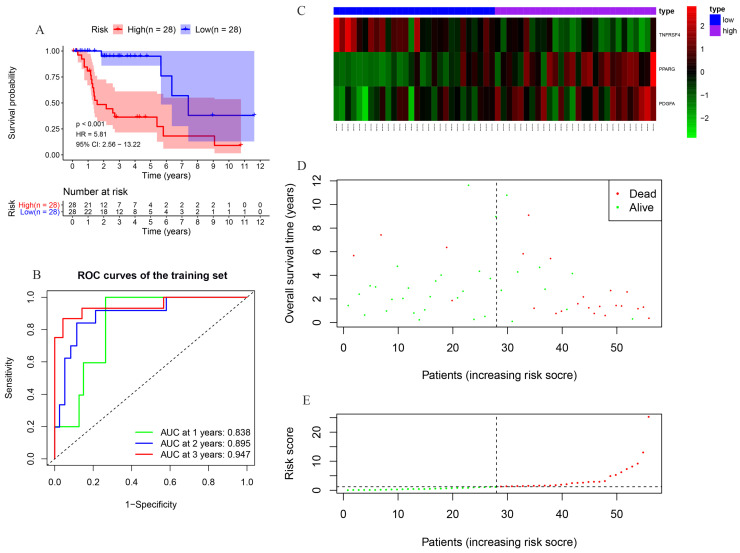
Prognostic performance in the training cohort. (**A**) Kaplan–Meier survival curves for low- and high-risk groups. (**B**) Time-dependent ROC curves. (**C**) Expression heatmap for the three signature genes. (**D**) Survival status and follow-up time. (**E**) Ordered risk-score distribution. In D and E, vertical dotted lines indicate the median risk-score cutoff separating low- and high-risk groups; in E, the horizontal dotted line indicates the risk-score cutoff.

**Figure 7:**
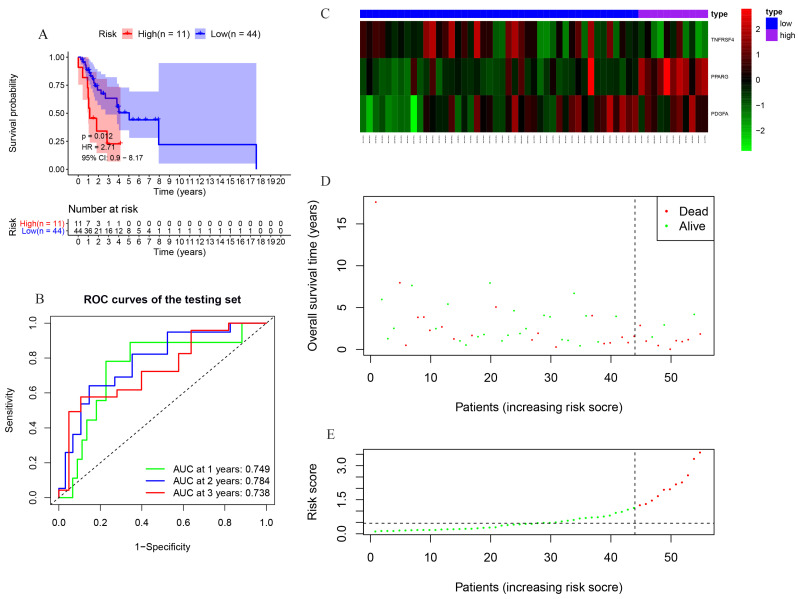
Internal validation in the testing cohort. (**A**) Kaplan–Meier curves for low- and high-risk groups. (**B**) Time-dependent ROC analysis. (**C**) Signature-gene expression heatmap. (**D**) Survival status and follow-up time. (**E**) Ordered risk-score distribution. In D and E, vertical dotted lines indicate the risk-score cutoff separating low- and high-risk groups; in E, the horizontal dotted line indicates the risk-score cutoff.

**Figure 8:**
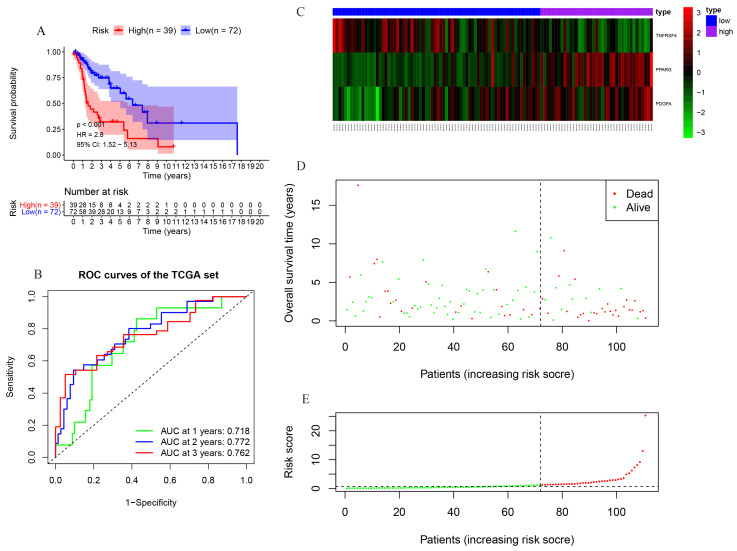
Model assessment using the full TCGA cohort. (**A**) Kaplan–Meier survival curves. (**B**) Time-dependent ROC analysis. (**C**) Signature-gene expression heatmap. (**D**) Survival status and follow-up time. (**E**) Ordered risk-score distribution. In D and E, vertical dotted lines indicate the risk-score cutoff separating low- and high-risk groups; in E, the horizontal dotted line indicates the risk-score cutoff.

The prognostic value of the three-gene signature (TNFRSF4, PPARG, and PDGFA) was evaluated by stratifying patients into high- and low-risk groups using the median risk score of the training cohort (*n* = 28 each). Kaplan–Meier analysis showed poorer overall survival in the high-risk group (*p* < 0.001; HR = 5.81, 95% CI: 2.56–13.22; Figure 6A). The apparent 1-, 2-, and 3-year AUC values in the training cohort were 0.838, 0.895, and 0.947, respectively (Figure 6B). Because of the limited number of patients and events, these training-set estimates may be optimistic. In the testing cohort and the full TCGA cohort, predictive performance decreased but remained evaluable (Figure 7 and Figure 8). External validation was performed in GSE27020 after probe-to-gene mapping; the Kaplan–Meier analysis results are shown in Supplementary Figure S1. The apparent 1-, 2-, and 3-year AUC values in the GSE27020 cohort were 0.719, 0.753, and 0.725, respectively (Supplementary Figure S1). These findings suggest that the signature has prognostic potential, although further validation in larger cohorts is necessary.

### 3.6. Verification by qRT-PCR

**Figure 9:**
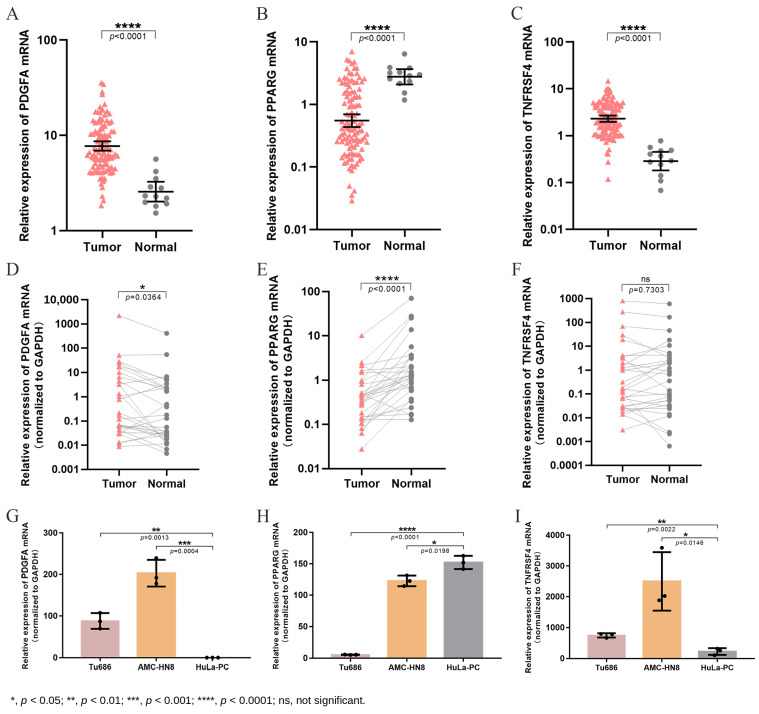
Expression of PDGFA, PPARG and TNFRSF4. (**A**–**C**) Expression in TCGA tumor and adjacent normal samples. (**D**–**F**) Expression in paired institutional LSCC and adjacent normal tissues. (**G**–**I**) Expression in AMC-HN8 and Tu686 LSCC cells compared with HuLa-PC laryngeal epithelial cells.

To validate the expression of the three-gene signature, we assessed the expression of PDGFA, PPARG, and TNFRSF4 in our institutional cohort. TCGA analysis showed higher PDGFA and TNFRSF4 expression and lower PPARG expression in tumor tissues compared with normal samples (Figure 9A–C). In paired institutional tumor-normal tissues, PDGFA was upregulated and PPARG was downregulated in tumors, whereas TNFRSF4 showed no significant difference (*p* = 0.7303; Figure 9D–F), possibly reflecting cohort-specific heterogeneity. Analysis of three LSCC cell lines revealed consistent differential expression patterns, further supporting the clinical significance of these genes (Figure 9G–I). Overall, qRT-PCR provided partial support for the three-gene signature but also highlighted the need for further validation of TNFRSF4.

### 3.7. Independent Prognosis Analysis

To assess the independence of the prognostic signature, we performed Cox regression analyses using the TCGA cohort and available clinical variables. Both univariate and multivariate analyses showed that risk score and gender were significantly associated with overall survival, whereas age, tumor grade, and stage were not statistically significant (Table 3). The lack of significance for stage and grade may reflect a limited sample size, imbalanced stage distribution, missingness, and the predominance of advanced-stage patients in the cohort. Specifically, the risk score (HR = 1.21, 95% CI: 1.11–1.32, *p* < 0.0001) remained independent prognostic factors after adjustment for clinical variables. These results highlight the robust and independent predictive value of our three-gene signature.

### 3.8. Construction of a Nomogram

**Figure 10:**
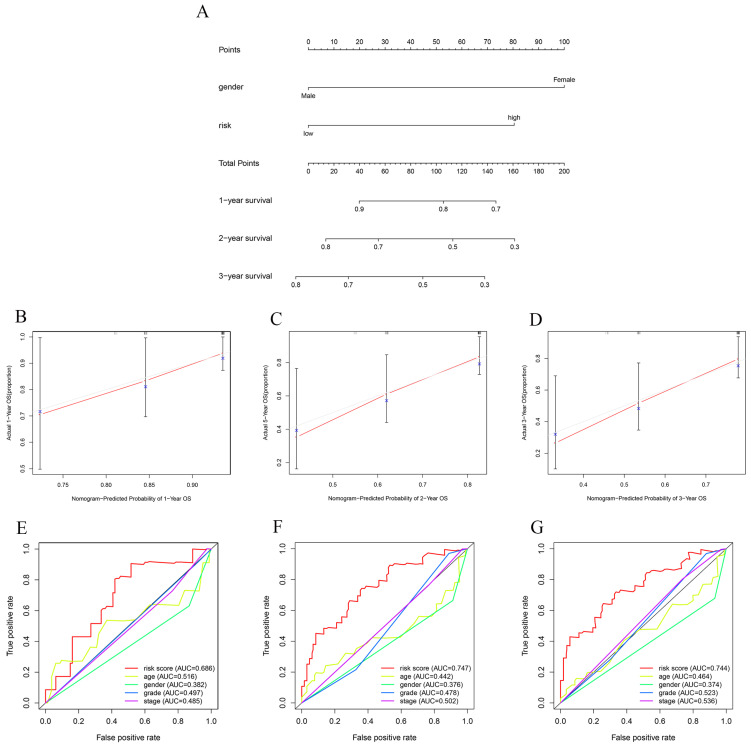
Exploratory nomogram and comparative discrimination. (**A**) Nomogram estimating 1-, 2- and 3-year overall survival in the TCGA cohort. (**B**–**D**) Calibration plots; red curves indicate nomogram-predicted calibration, and gray diagonal lines indicate ideal calibration. (**E**–**G**) ROC curves comparing the risk score with available clinicopathological variables.

To explore potential clinical applicability, we developed a nomogram incorporating the three-gene risk score and gender, which were significant in the multivariate model (Figure 10A). The nomogram estimates 1-, 2-, and 3-year survival probabilities. Calibration plots showed acceptable concordance between predicted and observed survival at all timepoints (Figure 10B–D). Time-dependent ROC analyses suggested that the risk score had higher AUCs than available clinicopathological parameters (age, gender, grade, and stage) in predicting 1-year (AUC = 0.686), 2-year (AUC = 0.747), and 3-year (AUC = 0.744) survival (Figure 10E–G), with particularly strong predictive value at 2 and 3 years.

## 4. Discussion

In this study, we have developed and validated a novel three-gene immune-related prognostic signature for LSCC, addressing a key gap in current prognostic assessment. Using comprehensive bioinformatic analysis of differentially expressed immune-related genes, followed by experimental validation, we identified TNFRSF4, PPARG, and PDGFA as independent predictors of patient outcomes. This signature demonstrated promising prognostic performance, effectively stratifying patients into high- and low-risk groups with significantly different survival outcomes (HR = 5.81, 95% CI: 2.56–13.22, *p* < 0.001). Importantly, its predictive accuracy was examined across the training, testing, entire TCGA, and GSE27020 datasets, supporting the reproducibility of the prognostic signature. Furthermore, comparative analysis showed that this three-gene signature achieved higher predictive performance than the available clinicopathological parameters in the current datasets, particularly in predicting both short-term and long-term survival. These results suggest that incorporating immune-related molecular features into prognostic models may provide a more precise tool for risk stratification and individualized prognostic assessment in LSCC, thereby enhancing clinical decision-making beyond the capacity of traditional staging systems.

Compared with previous immune-related prognostic signature study [18], our work focused on LSCC overall survival and proposed a compact three-gene model that was additionally assessed using qRT-PCR. In addition, we integrated the risk score with clinicopathological variables to construct and evaluate a prognostic nomogram. We believe that our work provides additional evidence supporting the prognostic relevance of immune-related molecular signatures in LSCC.

TNFRSF4 (also known as OX40), a member of the tumor necrosis factor receptor superfamily, demonstrated a protective effect (HR = 0.31) in our analysis. This finding is consistent with its established role in promoting T cell-mediated antitumor immunity [19,20]. However, TNFRSF4 did not show significant differential expression in our paired institutional qRT-PCR cohort. Therefore, its favorable prognostic association may be context dependent and may partly reflect differences in immune-cell infiltration or activation rather than tumor cell-intrinsic expression alone. Conversely, PPARG (HR = 3.37) and PDGFA (HR = 2.75) were associated with poorer prognosis, aligning with evidence supporting their involvement in immune suppression, tumor proliferation, and angiogenesis. PPARG is a central regulator of adipogenesis and plays a crucial role in modulating mitochondrial function [21,22]. An unresolved finding in the present study is the relationship between PPARG expression and clinical outcome. Although PPARG was downregulated in LSCC tumors relative to adjacent normal tissues, higher PPARG expression within the tumor cohort was associated with worse overall survival. While differential expression analyses and prognostic analyses address distinct biological questions, our data do not explain the mechanism underlying this association. Because the study relies primarily on bulk transcriptomic data, the cellular origin and functional significance of PPARG expression cannot be determined. Therefore, the biological basis of the observed prognostic effect remains unresolved and warrants further investigation. Notably, PPARG activity has been shown to drive macrophage polarization toward the tumor-promoting M2 phenotype [23]. Recent studies [24] also indicate that targeting the PPARG/CCL2 axis can modulate macrophage polarization and suppress tumor progression; for example, ginsenoside RK3 inhibits glioblastoma growth by downregulating PPARG, thereby reducing CCL2 secretion and attenuating M2 macrophage polarization. These findings underscore the possible therapeutic relevance of modulating PPARG signaling in the tumor microenvironment, although direct functional evidence in LSCC remains lacking. PDGFA (Platelet-Derived Growth Factor A) is a key mediator within the tumor microenvironment, exerting its effects on both fibroblasts and endothelial cells [25]. PDGFA regulates cancer-associated fibroblasts (CAFs), which are instrumental in extracellular matrix (ECM) remodeling and tumor progression. Elevated PDGFA expression has been reported in multiple cancer types and correlates with poor prognosis, likely due to its role in promoting stromal remodeling and facilitating immune evasion [26,27]. In LSCC, the prognostic signal of PDGFA may therefore reflect tumor-stromal interactions captured by bulk RNA-seq, and should be validated by cell-type-resolved approaches.

In summary, our results underscore both the prognostic significance and potential biological relevance of the TNFRSF4, PPARG, and PDGFA signature in LSCC. This three-gene model enables effective risk stratification and highlights hypothesis-generating targets for further investigation. Specifically, enhancing TNFRSF4-mediated immunity or inhibiting PPARG and PDGFA-driven tumor-promoting pathways may provide novel strategies for treatment. However, these therapeutic implications remain speculative and require mechanistic, preclinical, and clinical validation before they can be translated into patient management practices.

Immune-related genes play a pivotal role in modulating the tumor microenvironment (TME), influencing immune evasion and tumor progression in LSCC. The TME is increasingly recognized as a dynamic milieu where immune cells, stromal components, and tumor cells interact to either suppress or promote tumor growth [28,29]. The enrichment analysis revealed that DEIRGs are predominantly involved in pathways such as cytokine-cytokine receptor interaction, T cell activation, and leukocyte migration. These findings underscore the central role of immune signaling in LSCC, where dysregulated cytokine signaling can promote chronic inflammation and tumor progression [30]. T cell activation and leukocyte migration are essential for antitumor immune responses, but their impairment within the TME may contribute to immune escape, a hallmark of cancer [31,32]. Notably, dysregulated cytokine networks have been shown to orchestrate an immunologically “cold” microenvironment characterized by exclusion of effector cells, while compromised T cell receptor signaling and co-stimulatory pathways prevent development of robust antitumor responses even in the presence of tumor-reactive lymphocytes [33,34]. Further mechanistic insights have revealed that aberrant activation of PI3K-Akt and JAK-STAT signaling cascades serves as a molecular foundation for these immunosuppressive processes, concurrently promoting tumor growth, angiogenesis, and immune evasion across various malignancies, including head and neck squamous cell carcinoma [35,36]. This intricate network of immune dysregulation and oncogenic signaling underscores the complex interplay between host immunity and LSCC pathobiology, thereby providing rational targets for therapeutic intervention aimed at restoring immunological surveillance and control.

This study offers several notable strengths enhancing its translational impact. By integrating rigorous bioinformatics analysis with experimental validation, we ensured robust identification of differentially expressed immune-related genes and subsequent development of our three-gene signature. Because the cross-validated λ value was close to zero, the resulting model may remain susceptible to optimism and overfitting despite the use of LASSO selection, and the reported training-set performance should therefore be interpreted with caution. The signature’s prognostic value was assessed across multiple cohorts, supporting its preliminary reliability. As an OS-focused immune-related prognostic model developed specifically for LSCC, our work addresses a significant knowledge gap in this malignancy. The clinically applicable nomogram incorporating both molecular and clinical parameters demonstrated favorable predictive accuracy compared to conventional prognostic factors, providing a practical tool for individualized risk assessment that could enhance clinical decision-making for LSCC patients.

Despite the promising findings, this study has several limitations that warrant consideration. First, the TCGA training and testing cohorts were derived from a single dataset and therefore represent internal evaluation rather than independent validation. In addition, the external validation cohort (GSE27020) was generated using a microarray platform, whereas the discovery cohort was based on RNA sequencing. Platform-specific differences in transcript quantification, dynamic range, and signal distribution may affect model transferability. Consequently, the external validation results should be interpreted cautiously, and further validation in independent LSCC cohorts generated using harmonized transcriptomic platforms is warranted. Second, potential heterogeneity in gene expression patterns across LSCC subtypes and varying patient demographics may impact the reproducibility of the findings. LSCC is known to have distinct molecular and clinical subtypes, and further stratification by these subtypes could provide deeper insights into the applicability of the prognostic model. The third limitation is the lack of in vivo or in vitro functional studies to confirm the mechanistic roles of TNFRSF4, PPARG, and PDGFA in LSCC progression and immune modulation. Fourth, most patients with available staging information had stage III-IV disease, indicating a predominance of advanced disease. Furthermore, treatment-related information in TCGA was incomplete and inconsistently annotated, precluding robust adjustment for treatment modality. Consequently, residual confounding related to disease severity and treatment exposure cannot be excluded, and the prognostic contribution of the signature should be interpreted within this context. Finally, this study did not include immune infiltration quantification, PD-L1 expression, tumor mutational burden, cytolytic activity, single-cell or spatial analyses, or immunotherapy-treated cohorts; therefore, the current data support prognostic stratification but not prediction of immunotherapy response.

Future research should advance this work through three complementary approaches. First, mechanistic investigations should elucidate how TNFRSF4, PPARG, and PDGFA functionally interact within the tumor immune microenvironment, particularly focusing on TNFRSF4’s role in T cell activation versus the immunosuppressive and stromal remodeling functions of PPARG and PDGFA. Second, although treatment-related annotations were reviewed, the available TCGA data were too incomplete and heterogeneous to support meaningful adjustment or subgroup analysis. Therefore, potential confounding by treatment modality remains largely unexamined in the present study, which is needed to confirm the signature’s prognostic utility across diverse populations and potentially expand its application to treatment response prediction. Integration with complementary biomarkers could further enhance its clinical value. Finally, therapeutic targeting of these genes merits exploration, with TNFRSF4 agonism potentially enhancing antitumor immunity while PPARG/PDGFA inhibition may reverse immunosuppression and disrupt tumor-stromal interactions.

## 5. Conclusions

In conclusion, we identified and validated a novel three-gene immune-related prognostic signature (TNFRSF4, PPARG, and PDGFA) for laryngeal squamous cell carcinoma. This signature effectively stratifies patients by risk and showed potential prognostic value compared to traditional clinicopathological factors. Functional analyses highlight the involvement of these genes in key immune regulatory pathways and suggest their possible biological relevance. Our findings offer a practical tool for individualized prognosis and follow-up stratification in LSCC.

## Figures and Tables

**Table 1:** Univariate Cox regression analysis of prognosis-related immune genes.

id	HR	HR.95L	HR.95H	*p*-Value
ULBP1	2.868292451	1.629507305	5.048827679	0.000259734
MAVS	2.852556134	1.370873069	5.935689219	0.005051906
TNFRSF4	0.210922639	0.069850894	0.636904655	0.005779074
PPARG	2.849598667	1.335317942	6.081107959	0.006775899
PDGFA	2.694393362	1.299668831	5.585850345	0.007707973
IL13RA1	2.418779703	1.250603047	4.678139291	0.008679718
THRB	2.497970957	1.24771413	5.001032491	0.009742484
IREB2	3.393891273	1.259332186	9.14651281	0.01569991
CX3CR1	7.405093554	1.379485129	39.75063549	0.019533833
RORA	2.23360735	1.115858958	4.470996765	0.023234082
PSMD6	3.619142085	1.180156221	11.09869117	0.024468763
AHNAK	1.797458569	1.075864137	3.003034672	0.025142525
ACVR2A	4.083824128	1.190178404	14.01270553	0.025303847
TK2	4.018283071	1.174039607	13.75302737	0.026722169
STC2	1.725751723	1.056423912	2.819151456	0.029318879
ACVR1B	2.65215381	1.09218341	6.440236838	0.031180125
GAL	1.665160178	1.042228851	2.660412267	0.032926578
RNASEL	2.800807035	1.067829229	7.346230873	0.036317298
HSPA4	2.604997479	1.061680131	6.391766854	0.036558643
CMTM6	2.131333928	1.03696991	4.380632715	0.039520522
CXCL9	0.709446883	0.511366421	0.98425485	0.039881737
RARRES3	0.62307256	0.396114101	0.980069665	0.040650853
CASP3	2.027816269	1.030012818	3.992221017	0.040803047
BTC	2.39008611	1.016060661	5.622215123	0.045882648
TFRC	1.417583052	1.005038568	1.999467258	0.046744759
RASGRP1	2.322390741	1.011575611	5.331780142	0.046910642

**Table 2:** Multivariable Cox regression analysis of the three-gene signature.

id	Coef	HR	HR.95L	HR.95H	*p*-Value
TNFRSF4	−1.175739543	0.308590679	0.097436405	0.977337036	0.045615082
PPARG	1.214802258	3.369627683	1.414830635	8.025264962	0.006074962
PDGFA	1.01188154	2.750771835	1.288909607	5.870656599	0.008893179

**Table 3:** Cox regression analysis of the prognostic signature and clinicopathological variables.

		Univariate Analysis			Multivariate Analysis	
	HR	95% CI	*p*-Value	HR	95% CI	*p*-Value
age	1.01	0.97–1.06	0.626157165	1.02	0.97–1.06	0.480244125
gender	0.29	0.13–0.64	0.002147636	0.24	0.10–0.56	0.000983792
grade	0.82	0.49–1.39	0.465253841	0.85	0.47–1.53	0.58926013
stage	1.17	0.68–1.99	0.577492634	1.48	0.82–2.67	0.197730571
risk score	1.21	1.11–1.33	1.46902 × 10^−5^	1.21	1.11–1.32	2.55495 × 10^−5^

## Data Availability

The TCGA and GEO datasets analyzed in this study are publicly available from the TCGA data portal (https://tcga-data.nci.nih.gov/tcga/, accessed on 15 March 2023) and Gene Expression Omnibus (https://www.ncbi.nlm.nih.gov/geo/, accessed on 15 March 2023). Additional raw experimental data are available from the corresponding authors upon reasonable request.

## Supplementary Materials

The following supporting information can be downloaded at: https://www.mdpi.com/article/10.3390/jcm15145382/s1, Figure S1: External validation of the three-gene signature in the GSE27020 cohort; Table S1: Clinical information of TCGA cohort; Table S2: Enrichment analysis of subnetworks; Table S3: The sequences of all primers used in this study; Table S4: Clinical information of GSE27020 cohort; Table S5: Clinical information of qRT-PCR cohort.

## Abbreviations

LSCC, laryngeal squamous cell carcinoma; IRGs, immune-related genes; TCGA, The Cancer Genome Atlas; GEO, Gene Expression Omnibus; DEIRGs, differentially expressed immune-related genes; GO, Gene Ontology; KEGG, Kyoto Encyclopedia of Genes and Genomes; LASSO, least absolute shrinkage and selection operator; qRT-PCR, quantitative reverse-transcription PCR; RNA-seq, RNA sequencing; BP, biological process; CC, cellular component; MF, molecular function; OS, overall survival; ROC, receiver operating characteristic; TNFRSF4, tumor necrosis factor receptor superfamily member 4; PPARG, peroxisome proliferator-activated receptor gamma; PDGFA, platelet-derived growth factor subunit A.

## References

[1] Marur S., Forastiere A.A. (2016). Head and Neck Squamous Cell Carcinoma: Update on Epidemiology, Diagnosis, and Treatment. Mayo Clin. Proc..

[2] Li Y., Liu J., Hu W., Zhang Y., Sang J., Li H., Ma T., Bo Y., Bai T., Guo H. (2019). miR-424-5p Promotes Proliferation, Migration and Invasion of Laryngeal Squamous Cell Carcinoma. Onco Targets Ther..

[3] Jou A., Hess J. (2017). Epidemiology and Molecular Biology of Head and Neck Cancer. Oncol. Res. Treat..

[4] Liu B., Song Y., Liu D. (2017). Recent development in clinical applications of PD-1 and PD-L1 antibodies for cancer immunotherapy. J. Hematol. Oncol..

[5] Dine J., Gordon R., Shames Y., Kasler M.K., Barton-Burke M. (2017). Immune Checkpoint Inhibitors: An Innovation in Immunotherapy for the Treatment and Management of Patients with Cancer. Asia Pac. J. Oncol. Nurs..

[6] Le X., Ferrarotto R., Wise-Draper T., Gillison M. (2020). Evolving Role of Immunotherapy in Recurrent Metastatic Head and Neck Cancer. J. Natl. Compr. Cancer Netw..

[7] Guo Z., Li K., Ren X., Wang X., Yang D., Ma S., Zeng X., Zhang P. (2025). The role of the tumor microenvironment in HNSCC resistance and targeted therapy. Front. Immunol..

[8] Jiang P., Li Y., Xu Z., He S. (2021). A signature of 17 immune-related gene pairs predicts prognosis and immune status in HNSCC patients. Transl. Oncol..

[9] Zhang F., Liu Y., Yang Y., Yang K. (2020). Development and validation of a fourteen- innate immunity-related gene pairs signature for predicting prognosis head and neck squamous cell carcinoma. BMC Cancer.

[10] Tomczak K., Czerwinska P., Wiznerowicz M. (2015). The Cancer Genome Atlas (TCGA): An immeasurable source of knowledge. Contemp. Oncol..

[11] Edgar R., Domrachev M., Lash A.E. (2002). Gene Expression Omnibus: NCBI gene expression and hybridization array data repository. Nucleic Acids Res..

[12] Fountzilas E., Kotoula V., Angouridakis N., Karasmanis I., Wirtz R.M., Eleftheraki A.G., Veltrup E., Markou K., Nikolaou A., Pectasides D. (2013). Identification and validation of a multigene predictor of recurrence in primary laryngeal cancer. PLoS ONE.

[13] Kanehisa M., Furumichi M., Sato Y., Matsuura Y., Ishiguro-Watanabe M. (2025). KEGG: Biological systems database as a model of the real world. Nucleic Acids Res..

[14] Kanehisa M. (2019). Toward understanding the origin and evolution of cellular organisms. Protein Sci..

[15] Kanehisa M., Goto S. (2000). KEGG: Kyoto encyclopedia of genes and genomes. Nucleic Acids Res..

[16] Szklarczyk D., Gable A.L., Lyon D., Junge A., Wyder S., Huerta-Cepas J., Simonovic M., Doncheva N.T., Morris J.H., Bork P. (2019). STRING v11: Protein-protein association networks with increased coverage, supporting functional discovery in genome-wide experimental datasets. Nucleic Acids Res..

[17] Shannon P., Markiel A., Ozier O., Baliga N.S., Wang J.T., Ramage D., Amin N., Schwikowski B., Ideker T. (2003). Cytoscape: A software environment for integrated models of biomolecular interaction networks. Genome Res..

[18] Zhang H., Zhao X., Wang J., Ji W. (2021). Development and Validation of an Immune-Related Signature for the Prediction of Recurrence Risk of Patients With Laryngeal Cancer. Front. Oncol..

[19] Buchan S.L., Rogel A., Al-Shamkhani A. (2018). The immunobiology of CD27 and OX40 and their potential as targets for cancer immunotherapy. Blood.

[20] Jahan N., Talat H., Curry W.T. (2018). Agonist OX40 immunotherapy improves survival in glioma-bearing mice and is complementary with vaccination with irradiated GM-CSF-expressing tumor cells. Neuro Oncol..

[21] Liu C., Tate T., Batourina E., Truschel S.T., Potter S., Adam M., Xiang T., Picard M., Reiley M., Schneider K. (2019). Pparg promotes differentiation and regulates mitochondrial gene expression in bladder epithelial cells. Nat. Commun..

[22] Zhao M., Li X., Zhang Y., Zhu H., Han Z., Kang Y. (2020). PPARG Drives Molecular Networks as an Inhibitor for the Pathologic Development and Progression of Lung Adenocarcinoma. PPAR Res..

[23] Mehla K., Singh P.K. (2019). Metabolic Regulation of Macrophage Polarization in Cancer. Trends Cancer.

[24] Zhang H., Hu J., Zhao X., Zheng B., Han Y., Luo G., Dou D. (2025). Ginsenoside RK3 inhibits glioblastoma by modulating macrophage M2 polarization via the PPARG/CCL2 axis. Phytomedicine.

[25] Wang K., Li C., Chen H., Gu P., Lu J., Zhao H., Zhuo X. (2024). Increased Expression of PDGFA Is Associated With Poor Prognosis and Immune Infiltration in Head and Neck Squamous Cell Carcinoma. J. Oral Pathol. Med..

[26] Sahraei M., Roy L.D., Curry J.M., Teresa T.L., Nath S., Besmer D., Kidiyoor A., Dalia R., Gendler S.J., Mukherjee P. (2012). MUC1 regulates PDGFA expression during pancreatic cancer progression. Oncogene.

[27] Martinho O., Longatto-Filho A., Lambros M.B., Martins A., Pinheiro C., Silva A., Pardal F., Amorim J., Mackay A., Milanezi F. (2009). Expression, mutation and copy number analysis of platelet-derived growth factor receptor A (PDGFRA) and its ligand PDGFA in gliomas. Br. J. Cancer.

[28] Hanahan D., Weinberg R.A. (2011). Hallmarks of cancer: The next generation. Cell.

[29] Vitale I., Shema E., Loi S., Galluzzi L. (2021). Intratumoral heterogeneity in cancer progression and response to immunotherapy. Nat. Med..

[30] West N.R., McCuaig S., Franchini F., Powrie F. (2015). Emerging cytokine networks in colorectal cancer. Nat. Rev. Immunol..

[31] Diehl A.D., Lee J.A., Scheuermann R.H., Blake J.A. (2007). Ontology development for biological systems: Immunology. Bioinformatics.

[32] Lieber S., Reinartz S., Raifer H., Finkernagel F., Dreyer T., Bronger H., Jansen J.M., Wagner U., Worzfeld T., Muller R. (2018). Prognosis of ovarian cancer is associated with effector memory CD8(+) T cell accumulation in ascites, CXCL9 levels and activation-triggered signal transduction in T cells. Oncoimmunology.

[33] Dyck L., Mills K.H.G. (2017). Immune checkpoints and their inhibition in cancer and infectious diseases. Eur. J. Immunol..

[34] Darvin P., Toor S.M., Sasidharan Nair V., Elkord E. (2018). Immune checkpoint inhibitors: Recent progress and potential biomarkers. Exp. Mol. Med..

[35] Wang S.C., Chai D.S., Chen C.B., Wang Z.Y., Wang L. (2015). HPIP promotes thyroid cancer cell growth, migration and EMT through activating PI3K/AKT signaling pathway. Biomed. Pharmacother..

[36] Slattery M.L., Lundgreen A., Kadlubar S.A., Bondurant K.L., Wolff R.K. (2013). JAK/STAT/SOCS-signaling pathway and colon and rectal cancer. Mol. Carcinog..

