# Supplementary figures and images for: Development and Validation of an Immune-Related Prognostic Signature for Laryngeal Squamous Cell Carcinoma
Source: J Clin Med. 2026 Jul 9;15(14):5382. doi: 10.3390/jcm15145382 (PMC13412633; doi:10.3390/jcm15145382)

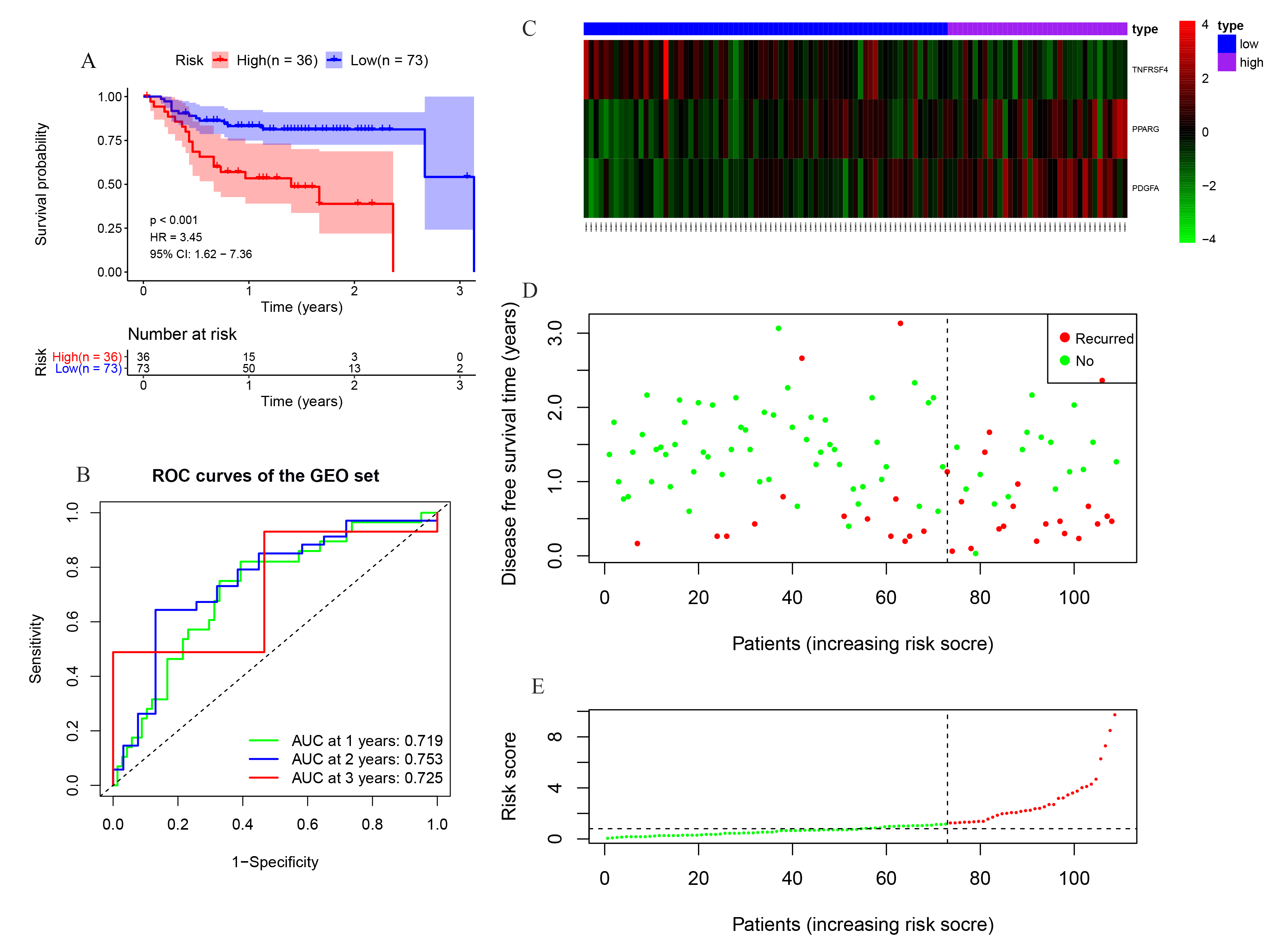

Supplement: Supplementary file 1 [file jcm-15-05382-s001.zip › Supplementary Figure S1 External validation of the three-gene signature in the GSE27020 cohort.tif]
